# Efficient Recovery of Organic Matter from Municipal Wastewater by a High-Rate Membrane Bioreactor Equipped with Flat-Sheet Ceramic Membranes

**DOI:** 10.3390/membranes13030300

**Published:** 2023-03-03

**Authors:** Michael Joseph Rocco, Akira Hafuka, Toru Tsuchiya, Katsuki Kimura

**Affiliations:** 1 Division of Environmental Engineering, Hokkaido University, N13W8, Kita-ku, Sapporo 060-8628, Japan; 2 MEIDENSHA CORPORATION, ThinkPark Tower, 2-1-1 Osaki, Shinagawa-ku, Tokyo 141-6029, Japan

**Keywords:** wastewater treatment, carbon recovery, membrane fouling, granular scouring, chemically enhanced backwash

## Abstract

High-rate processes have been investigated for the recovery of organic matter from municipal wastewater. High-rate membrane bioreactors (HR-MBRs) may simultaneously achieve the increased recovery of carbon and high effluent quality, although control of membrane fouling is extremely difficult. To address the severe fouling in HR-MBRs, the combination of granular scouring and frequent chemically enhanced backwashing was examined. The use of robust flat-sheet ceramic membranes enabled the application of those cleaning strategies. Experiments were carried out at an existing wastewater treatment plant. To operate as a high-rate system, the bioreactor solid residence time and hydraulic residence time were set at 0.5 days and 1.6 h, respectively. Although a relatively high flux of 20 L m^−2^ h^−1^ was applied, the proposed HR-MBR exhibited a very low fouling rate of 1.3 kPa/day. The system could recover >70% of the carbon from raw wastewater, whereas the concentration of chemical oxygen demand in the effluent was lowered to <20 mg/L. The performance of the proposed HR-MBR observed in this study was clearly superior to those reported in previous related studies.

## 1. Introduction

Wastewater treatment plants (WWTPs) are significant contributors to electricity consumption in cities. It has been estimated that WWTPs consume 1–3% and 3–4% of the total electricity in Europe and in the USA, respectively [1]. A reduction in energy consumption in WWTPs is required, and new options for converting wastewater treatment into a net-positive energy process have been investigated in recent studies. A high-rate activated sludge (HRAS) process is a biological wastewater treatment technology that is characterized by an extremely short (0.5 to 2 days) solid residence time (SRT) and an extremely short (less than 5 h) hydraulic residence time (HRT) [2]. HRAS processes enable the capture of colloidal and dissolved organic matter in raw wastewater into microbial flocs without degradation [3], leading to an increase in the amount of organic matter utilized in anaerobic digestion [4] and a reduction in net energy consumption in WWTPs.

Generally, in HRAS processes, a shorter SRT leads to higher carbon capture rates and a longer SRT leads to better effluent quality (i.e., a higher degree of mineralization of the organic carbon and a reduction in carbon capture) [5]. Due to this tradeoff, simultaneously achieving a high recovery rate of carbon and a sufficiently high-quality effluent would be difficult with an HRAS process. Membrane separation may address this tradeoff in an HRAS process by providing complete retention of particles, including biomass and colloidal matter. A combination of a membrane process and a biological treatment is known as a membrane bioreactor (MBR). Although many full-scale MBRs have been installed worldwide, MBRs have been rarely used as high-rate processes for capturing carbon. The short SRT that is necessary for high-rate processes causes extremely severe membrane fouling in high-rate MBRs (HR-MBRs) [6]. Commonly, MBRs are operated with a long SRT of >10 days [7], whereas HR-MBRs need to be operated with an SRT of only 0.5–2 days. The fouling in HR-MBRs could not be effectively controlled in previous studies [8,9,10]. The aforementioned problem of membrane fouling in HR-MBRs, however, may be addressed by intensive membrane cleaning. Very intensive membrane cleaning for conventional MBRs by using a combination of granular scouring and chemically enhanced backwashing (CEB) has been proposed [11,12]. The use of robust flat-sheet ceramic membranes enabled the implementation of such intensive cleaning. It was thought that this approach might also be effective for the HR-MBRs used for capturing carbon from municipal wastewater, in which extremely severe membrane fouling occurs. To the best of our knowledge, such an investigation has never been conducted.

The objective of this study was to demonstrate the feasibility of an HR-MBR in which granular scouring and frequent CEB were carried out. The feasibility of the HR-MBR was claimed based on a balanced result of high effluent water quality, low membrane fouling rate and a high recovery rate of organic matter. The recovery of nitrogen and phosphorus from wastewater was out of the scope of the present study but should be examined in future works.

## 2. Materials and Methods

### 2.1. Experimental Setup

**Figure 1:**
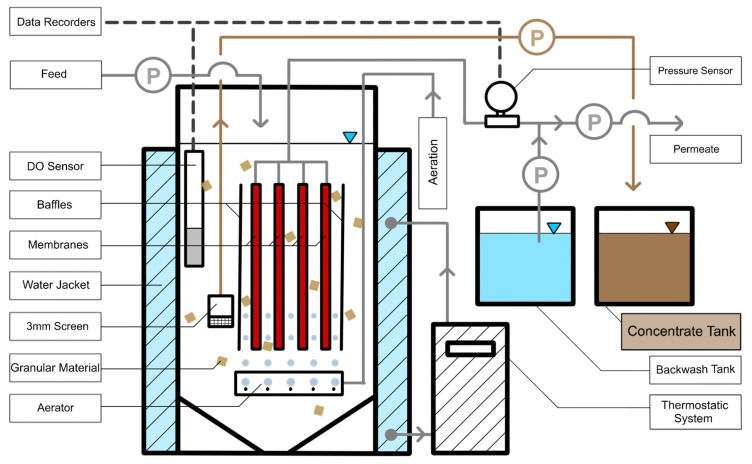
Schematic flow diagram of the experimental system. The shaded area indicates the closed loop water circulator for temperature control.

Bench-scale experiments were carried out at an existing wastewater treatment plant (Sapporo, Japan) connected to a combined sewer system. Flat-sheet ceramic membranes (Meidensha, Tokyo, Japan) with a nominal pore size of 0.1 µm and total membrane area of 0.2048 m^2^ were immersed in an 8.4 L reactor. Figure 1 shows a schematic flow diagram of the experimental setup. The granules used for scouring the membrane surface in this study were cylindrical granules (4 mm in diameter and length) made from polyethylene glycol (BCN, Nisshinbo Chemical Inc., Tokyo, Japan). Clearances between the membranes were 10 mm, allowing free movement of the granules for efficient scouring of the membrane surface. The granules were fluidized by a coarse bubble aeration flow (4 L/min) provided through two half-inch tubes with holes of 2 mm in diameter. The temperature inside the reactor was maintained at 19.4 ± 0.8 °C by a thermostatic system (MT-1000, Eyela, Tokyo, Japan). The effluent from the primary sedimentation basin of the WWTP was used as the feed in this study. The average feed properties are shown in Supporting Materials (Table S1). Concentrate (i.e., recovered organic matter) was withdrawn directly and continuously from the HR-MBR by using a peristaltic pump. A screen (3 mm holes) was installed in the concentrate recovery line to prevent the loss of granules.

### 2.2. Experimental Conditions

Prior to the initiation of each experiment, the membranes were thoroughly cleaned (a 24 h soak in a 2000 ppm sodium hypochlorite (NaClO) solution followed by a 24 h soak in a 2000 mg/L citric acid solution) to ensure that the filtration resistance of the membranes was the same as that of a new membrane. Filtration resistance was assessed for all membranes prior to use. The initial resistance of the membranes used was 4.5 ± 0.1 × 10^11^ m^−1^, which is close to the resistance of a new membrane (3.9 ± 0.1 × 10^11^ m^−1^). In all experiments, net filtration flux, aeration rate, and SRT were fixed at 20 L m^−2^ h^−1^ (LMH), 4 L/min, and 0.5 days, respectively. Based on the results obtained in preliminary experiments, granular materials were placed in the reactor so that the volume of the granules was 30% of the reactor’s total volume.

Two series of experiments, Runs 1 and 2, were conducted in this study. Run 1 was carried out to investigate concentrations of NaClO for CEB at which stable operation of the HR-MBR could be sustained. A NaClO concentration of 500–3000 mg/L is typically used to remove irreversible fouling in submerged MBRs [13]. In this study, 1000 mg/L and 500 mg/L were chosen as high concentrations of NaClO for CEB. A low concentration of 50 mg/L and an intermediate concentration of 100 mg/L were also investigated. Run 2 was conducted to investigate the extent of carbon recovery achievable by the proposed HR-MBR using ceramic membranes. In Run 2, the operation was carried out for 4 days, corresponding to 8 times the SRT. It should be noted that SRT in the HR-MBR was very short in this study (12 h). Therefore, the HR-MBR could be started up within a few days and no seed sludge was added.

### 2.3. Analytical Methods

Measurements of total organic carbon (TOC) were carried out using a TOC analyzer (TOC-L, Shimadzu, Kyoto, Japan). A commercially available analytical kit (HACH, Loveland, CO, USA, Method 8000) was used for chemical oxygen demand (COD) measurements. Amounts of suspended solids (SS) and volatile suspended solids (VSS) were determined using standard methods [14].

### 2.4. Assessments of Membrane Fouling

Clean water permeability measurements were conducted in this study to assess the filtration resistance caused by fouling on/in the membrane. Based on Darcy’s Law, filtration resistance of the membranes was calculated using Equation (1).

(1) $$J=\frac{\Delta P}{\mu R}=\frac{\Delta P}{\mu \left(R_{m}+R_{r}+R_{irr}\right)}$$

where *R* is the total filtration resistance (m^−1^), Δ*P* is transmembrane pressure (TMP) (Pa), *J* is the flux (m^3^ m^−2^ s^−1^) and *µ* (g m^−1^ s^−1^) is the permeate viscosity. Total resistance was separated into three sources of resistance: intrinsic resistance of the membrane (*R_m_*), reversible resistance (*R_r_*) mainly caused by the cake and gel layers, and irreversible resistance (*R_irr_*) from the intrapore foulants. Definitions and the protocol for measurements of each resistance are described elsewhere [15].

### 2.5. Fractionation of Concentrate and Bioflocculation Analysis

The quantity of organic matter adsorbed onto sludge flocs (i.e., bioflocculation) was also assessed in this study. The method used for this assessment was similar to other carbon redirection studies [6,10], where the difference in particulate COD between the influent and the concentrate was regarded as adsorption of colloidal and dissolved COD onto the flocs. COD in the tank was fractionated into particulate, colloidal and soluble fractions in the following manner. Mixed liquor samples were centrifuged at 4000 rpm for 5 min (CT-6E, Hitachi, Tokyo, Japan) and supernatants were obtained. The soluble fraction was obtained by filtering the supernatants through 0.45 μm PTFE membranes (Advantec, Tokyo, Japan). The particulate COD fraction was determined by the difference between the total COD and the supernatant COD, whereas the colloidal COD fraction was determined by the difference between the supernatant COD and the soluble COD.

## 3. Results and Discussion

### 3.1. Effectiveness of the Combination of Granular Scouring and CEB with Various Concentrations of NaClO (Run 1)

#### 3.1.1. Fouling Evolution in Run 1

**Figure 2:**
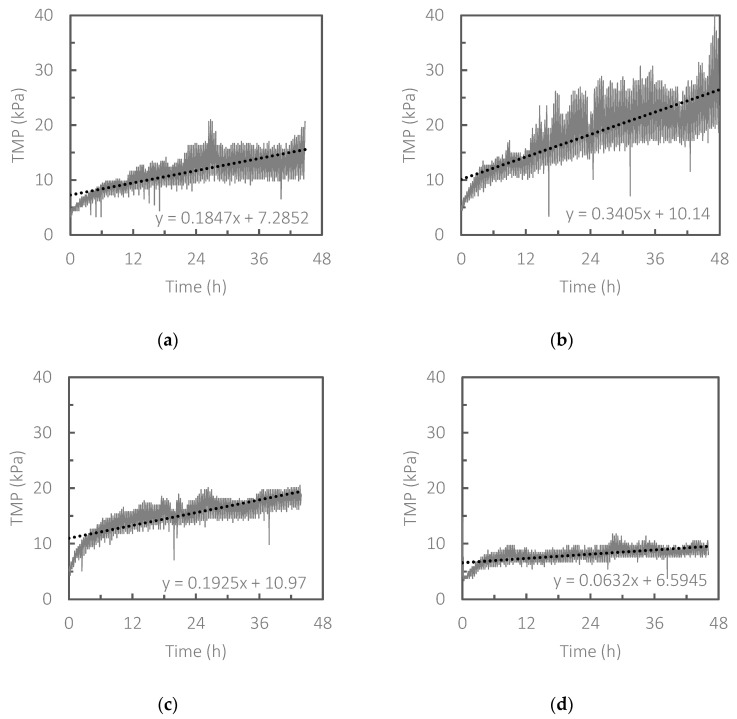
TMP evolution in Runs (**a**) 1.1, (**b**) 1.2, (**c**) 1.3 and (**d**) 1.4, in which the concentrations of NaClO in the backwash solution were set at 1000 ppm, 500 ppm, 100 ppm and 50 ppm, respectively. In these runs, granular scouring was also carried out.

In Run 1, the effectiveness of the combination of granular scouring and CEB for fouling control in the HR-MBR was examined with various CEB intensities (various NaClO concentrations in the backwash solution). Figure 2 shows the TMP increases in Run 1. The two control strategies could work in different ways: granular scouring and CEB were effective for removing foulants on the membrane surface and inside the micropores, respectively. Therefore, that combination could work complimentarily. Although TMP increased very rapidly and became uncontrollable within several hours in the control experiments in which either granular scouring or CEB was omitted (see Figure S1 in Supporting Material), the combination was effective for controlling membrane fouling in the HR-MBR to some extent, regardless of the CEB intensity. The slope of the linear regression line (dotted lines in Figure 2) of the TMP increase shows a rough estimation of the fouling rate in kPa per day. Fouling rates in Runs 1.1, 1.2, 1.3 and 1.4, calculated from the equation of the linear regression, were 4.43 kPa/d, 8.17 kPa/d, 4.62 kPa/d and 1.52 kPa/d, respectively. Interestingly, the lowest concentration of NaClO in the CEB tested, 50 ppm, exhibited the lowest fouling rate. Conventional MBRs treating municipal wastewater are normally operated at a fouling rate of less than 2 kPa/day [16,17]. It should be noted that the HR-MBR in this study was operated at a practical net flux of 20 LMH [18,19]. Therefore, the fouling rate of 1.52 kPa/d observed in Run 1.4 suggests that long-term operation of an HR-MBR is feasible, assuming that there is still plenty of room for further optimization of operational parameters.

#### 3.1.2. Analysis of Fouling Resistance in Run 1

**Figure 3:**
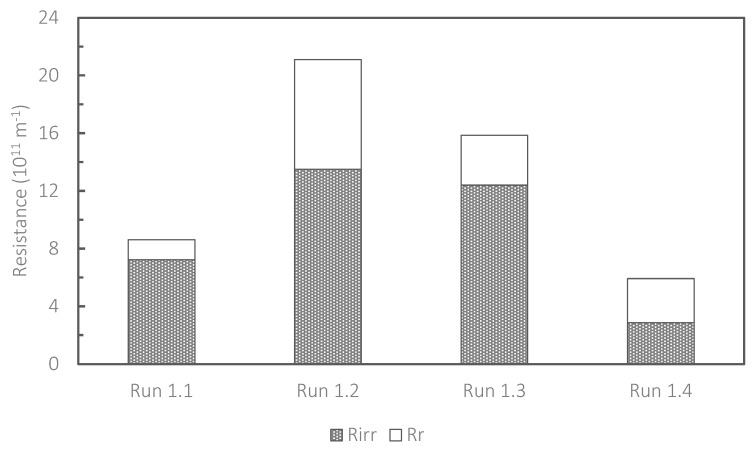
Distribution of fouling resistances assessed at the termination of Runs 1.1, 1.2, 1.3 and 1.4.

Figure 3 shows the distribution of fouling resistances assessed at the termination of Runs 1.1, 1.2, 1.3 and 1.4. The resistance occurring inside the membrane pores (i.e., irreversible fouling) was dominant in these runs except for Run 1.4. As mentioned before, the evolution of fouling was effectively controlled in Run 1.4, in which the lowest CEB intensity (50 ppm NaClO) was applied. This was largely attributed to the slowest evolution of irreversible fouling as shown in Figure 3. Kimura and Uchida (2019) showed that CEB using NaClO could induce the release of dissolved organic matter (DOM) from biomass in an aerobic MBR and that the released DOM caused fouling in ceramic membranes submerged in the MBR [11]. Similarly, Sun et al. (2018) reported that intensive in-line membrane cleaning in an MBR using ozonation induced the release of DOM from the mixed liquor suspension, which accelerated fouling of a crossflow microfiltration module [20]. It is thought that CEB with a high intensity (>100 ppm NaClO in the backwashing solution in this study) induces release of DOM from biomass in the HR-MBR, leading to the evolution of irreversible fouling. However, when the highest CEB (1000 ppm NaClO in Run 1.1) was examined, the degree of irreversible fouling was considerably lower. A possible explanation for this is that the high cleaning efficiency with 1000 ppm NaClO could offset the release of DOM from biomass to some extent. The degree of irreversible fouling with 1000 ppm NaClO was still higher than that with 50 ppm NaClO. Based on these observations, the lowest CEB concentration of 50 ppm was used in the subsequent test (Run 2). It should be noted that the degree of reversible fouling was the lowest with 1000 ppm NaClO. A sufficiently high CEB intensity may reduce the degree of reversible fouling, although economic feasibility and the evolution of irreversible fouling are potential problems. This point should be investigated in future studies.

#### 3.1.3. Bioflocculation in Run 1

**Figure 4:**
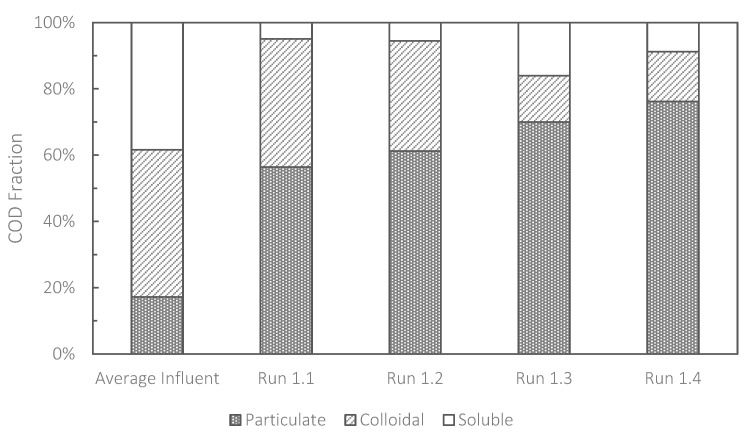
Size distributions (particulate, colloidal and soluble) of COD in the biomass suspension just before termination of Runs 1.1 to 1.4.

When bioflocculation occurs in HR-MBRs, adsorption of the soluble and colloidal organic matter onto the particulate fraction is promoted and the soluble and colloidal COD fractions in a mixed liquor suspension are subsequently reduced. Faust et al. (2014b) suggested that bioflocculation plays an important role in the maintenance of the high filterability of the mixed liquor in HR-MBRs [21]. Bioflocculation in HR-MBRs should, therefore, be maximized to carry out stable operation. Figure 4 shows the size distribution of organic matter (expressed as COD) in the HR-MBR in Run 1. The assessment, the results of which are shown in Figure 4, was carried out by the method used in previous studies [6,10]. Figure 4 shows that the presence of colloidal COD was substantial in Runs 1.1 and 1.2, indicating that bioflocculation was poor in those runs. High concentrations of NaClO in the CEB solution used in Runs 1.1 and 1.2 probably inhibited efficient bioflocculation by suppressing microbial activity. Sun et al. (2021) observed a reduction in the average particle size (i.e., deflocculation) after the implementation of CEB using NaClO in a conventional MBR (SRT of 30 days), which led to deterioration in the filterability of the mixed liquor [22]. The high concentration of colloidal COD could also explain the rapid evolution of fouling in Runs 1.1 and 1.2. It has been reported that the presence of colloidal organic matter in MBRs is closely related to the evolution of membrane fouling [23,24] and the reduced filterability of the mixed liquor [25]. In contrast, when the CEB intensity in an HR-MBR was reduced, bioflocculation could be promoted and high filterability of the mixed liquor was maintained. Enhanced bioflocculation is also beneficial as it increases the amount of carbon that can be utilized for biogas generation. Thus, the importance of a balance between the maintenance of bioflocculation and the cleaning efficiency of CEB for fouling control in HR-MBRs was demonstrated in this study.

### 3.2. Longer Operation of the HR-MBR (Run 2)

#### 3.2.1. Fouling Evolution in Run 2

**Figure 5:**
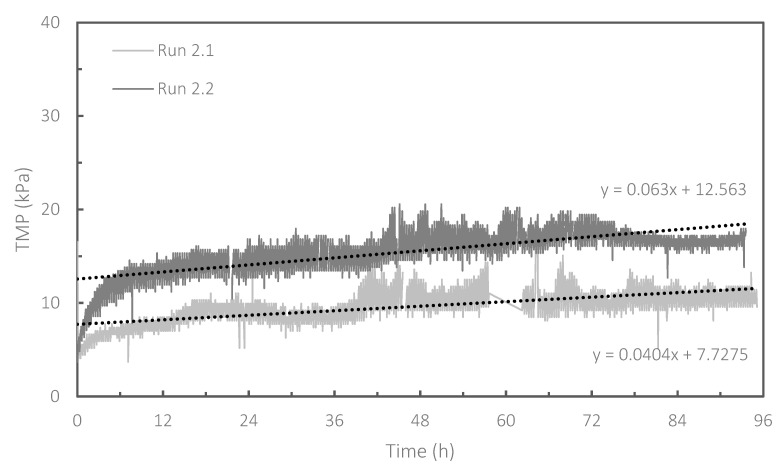
Increases in TMP in Runs 2.1 and 2.2. The dotted lines represent the linear regression of TMP evolutions. During Run 2.1, at around 2.5 days of operation, a power outage that lasted 4 h stopped all activity in the experimental system.

As stated in the previous section, it was demonstrated that the combination of granular scouring and CEB could be effective for the control of membrane fouling in the HR-MBR. It was also shown that the concentration of NaClO in the CEB solution had a significant impact on the evolution of fouling in the HR-MBR. Based on the results obtained in Run 1, long-term operations of the HR-MBR were attempted (Run 2). The concentration of NaClO in the CEB solution was set at 50 ppm based on the results of Run 1. Figure 5 shows the increases in TMP observed in Run 2. Operations lasting for 4 days were carried out twice (Run 2.1 and 2.2) under the same operational conditions as those tested in Run 1.4. In Runs 2.1 and 2.2, increases in TMP were relatively rapid during the first 5 h of the operation, possibly due to the absence of seeding at the beginning of the operations. After that, the rate of TMP increase became slower, and the operation could be carried out stably over a period of four days. Fouling rates in Runs 2.1 and 2.2 were 1.0 kPa/d and 1.5 kPa/d, respectively. Fouling rates in Runs 2.1 and 2.2 were slightly different. This was attributed to the difference in the quality of the influent wastewater. It should be noted that real municipal wastewater was used in this study: variation in the influent wastewater was uncontrollable.

#### 3.2.2. Carbon Recovery and Effluent Quality Achieved by the HR-MBR

By conducting longer-term operations (Run 2), it became possible to assess the carbon recovery achieved by the HR-MBR. On average, 58.9% of the COD in the primary sedimentation effluent could be recovered by the HR-MBR (see Figure S2 in Supporting Material). According to the annual report from the WWTP where this study was carried out, the primary treatment removed one-third of organic matter in the influent to the sedimentation basin. Thus, in total, 73.3% of organic matter in the raw wastewater could be recovered by combining primary sedimentation and the HR-MBR, and it could be used for methane generation. As discussed in the subsequent section, the carbon recovery achieved by the HR-MBR in this study was improved compared to that of previous related studies by implementing the primary sedimentation tank.

In Run 2, the average COD concentration in the permeate was 18.8 mg/L. About 95% of the COD in the influent to the WWTP was removed in the permeate of the HR-MBR. This removal efficiency is comparable to that reported for conventional MBRs (SRT of 15–60 days) treating municipal wastewater [19,26], indicating that the frequent CEB carried out in this study had a limited impact on microbial performance. The quality of the effluent obtained in this study with the HR-MBR was much higher than that found with other competitive technology for carbon recovery from municipal wastewater. It has been reported that HRAS systems (A-stage systems) can achieve a COD removal rate of 52–64% [27,28]. As another emerging technology for carbon capture, direct membrane filtration (DMF) (in which no biological treatment is promoted) could not produce treated water with a low concentration of COD (<30 mg/L) without using coagulation [29].

#### 3.2.3. Comparison with Other Reported HR-MBRs

Table 1 summarizes the performances of other HR-MBRs examined in recent studies and the performance of the HR-MBR used in this study. In this study, the HR-MBR exhibited COD removal of about 95%, which is better than the rates of removal reported for other HR-MBRs. The HR-MBR tested in this study recovered about 60% of organic matter contained in the feed (i.e., effluent of the primary sedimentation basin). The rate of carbon recovery achieved by the HR-MBR in this study was also comparable to the rates reported for other HR-MBRs. Compared with the other reported systems, the fouling rate observed in this study (1.3 kPa/day, Run 2) was considerably lower, even though the net flux set in this study was high (20 LMH). To conclude, membrane fouling in the HR-MBR tested in this study was more efficiently controlled than that in other reported HR-MBRs, while high rates of carbon recovery and COD removal were maintained.

## 4. Conclusions

The use of ceramic membranes enabled the application of granular scouring and frequent CEB, which are too intensive for polymeric membranes. Furthermore, the flat-sheet configuration promoted the efficient movements of granules. Thus, an HR-MBR using flat-sheet ceramic membranes is a promising technology. The performance of the HR-MBR observed in this study is characterized by stable membrane filtration at a relatively high flux of 20 LMH, high recovery of organic matter (>70%) and a low concentration of COD in the permeate (<20 mg/L), rates which are clearly superior to those of other HR-MBRs reported previously. The low SRT and HRT set in this study (0.5 days and 1.6 h, respectively) enabled efficient recovery of organic matter, whereas the severe fouling in the HR-MBR could be controlled by the intensive membrane cleaning. It should be noted that there is still plenty of room for optimization of the operating conditions of the HR-MBR. It is necessary to investigate how to incorporate processes of nutrient recovery in combination with the HR-MBR.

## Figures and Tables

**Table 1:** Summary of the performances of HR-MBRs in recent studies.

Reference	Membrane Type	Feedwater	Volume (L)	SRT (days)/HRT (hours)	MLVSS (mg/L)	COD Removal	Net Flux (Gross Flux) ^a^ (LMH)	Fouling Rate (kPa/d)	Carbon Recovery
Dai et al., 2018 [10]	Hollow-fiber polysulfone of 0.1 μm pores, 0.34 m^2^ (Dalian, China)	Screened and degritted WWTP influent with 441 ± 33 mg-COD/L	5.0	0.5/1.2	612 ± 122	Around 80%	11.0 (11.0)	4.5 ^b^	47.9%
Emaminejad et al., 2019 [30]	Flat sheet chlorinated polyethylene of 0.4 μm pores, 0.11 m^2^ (Kubota Co., Osaka, Japan)	Synthetic greywater with 387 ± 33 mg-COD/L	5.5	0.5/1.5	1297 ± 31	87%	25.0 (33.3)	N/A	54.3%
Wan et al., 2020 [31]	Hollow-fiber ultrafiltration polyvinylidene fluoride of 0.03 μm pores, 0.28 m^2^ (Tianjin, China)	Screened and degritted WWTP influent with 247 ± 21 mg-COD/L	1.7	0.6/1.0	N/A	86–89%	4.8 (6.0)	2.8–11.5 ^b^	65.1–67.1%
This Study	Flat sheet alumina ceramic of 0.1 μm pores, 0.2 m^2^ (Meidensha, Tokyo, Japan)	Effluent of the primary sedimentation basin with 209 ± 46 mg-COD/L	8.4	0.5/1.6	404 ± 108	94% ^c^	20.0 (25.0)	1.3	58.9% (73.3% ^d^)

^a^ Gross flux is the permeate flux during filtration and net flux is the total permeate flux considering relaxation and backwashing. ^b^ Estimated values from the data obtained in each study. ^c^ COD concentration in the influent to the WWTP was 339 mg/L. ^d^ Obtained from combining the carbon recovery of the primary sedimentation basin and the recovery from the HR-MBR. N/A = not available.

## Data Availability

Data will be made available on request.

## Supplementary Materials

The following supporting information can be downloaded at: https://www.mdpi.com/article/10.3390/membranes13030300/s1, Figure S1: The transmembrane pressure evolution during the operation of Runs (a) S1 without granular scouring and (b) S2 without CEB., Figure S2: Daily mass balances of the HR-MBR of Run (a) 2.1 and (b) 2.2.; Table S1: Average characteristics of the wastewater (primary sedimentation effluent) fed into the HR-MBR. Analysis was carried out for daily 24 h composite samples.
