# Supplementary material for: Efficient Recovery of Organic Matter from Municipal Wastewater by a High-Rate Membrane Bioreactor Equipped with Flat-Sheet Ceramic Membranes
Source: Membranes (Basel). 2023 Mar 3;13(3):300. doi: 10.3390/membranes13030300 (PMC10056867; doi:10.3390/membranes13030300)
Supplement: Supplementary file 1 [file membranes-13-00300-s001.zip › membranes-2220910-supplementary.pdf]

membranes

Supporting Materials

Efficient Recovery of Organic Matter from Municipal Wastewater by a High-Rate Membrane Bioreactor Equipped with Flat-Sheet Ceramic Membranes

Michael Joseph Rocco [1], Akira Hafuka [1], Toru Tsuchiya [2] and Katsuki Kimura [1,*]

[1] Division of Environmental Engineering, Hokkaido University, N13W8, Kita-ku, Sapporo 060-8628, Japan
[2] MEIDENSHA CORPORATION, ThinkPark Tower, 2-1-1 Osaki, Shinagawa-ku, Tokyo 141-6029, Japan
* Correspondence: kkatsu@eng.hokudai.ac.jp; Tel.: +81-11-7066271

A. Average primary sedimentation effluent properties

Table S1. Average characteristics of the wastewater (primary sedimentation effluent) fed into the HR-MBR. Analysis was carried out for daily 24-hour composite samples.

TOC (mg/L)	42.4 ± 11.4
T-P (mg/L)	5.6 ± 1.9
T-N (mg/L)	20.8 ± 4.4
pH	7.1 ± 0.2
Temperature (°C)	19.6 ± 3.2
SS (mg/L)	62.2 ± 16.2
VSS (mg/L)	41.4 ± 11.7

B Assessment of Organic Carbon Recovery by the HR-MBR

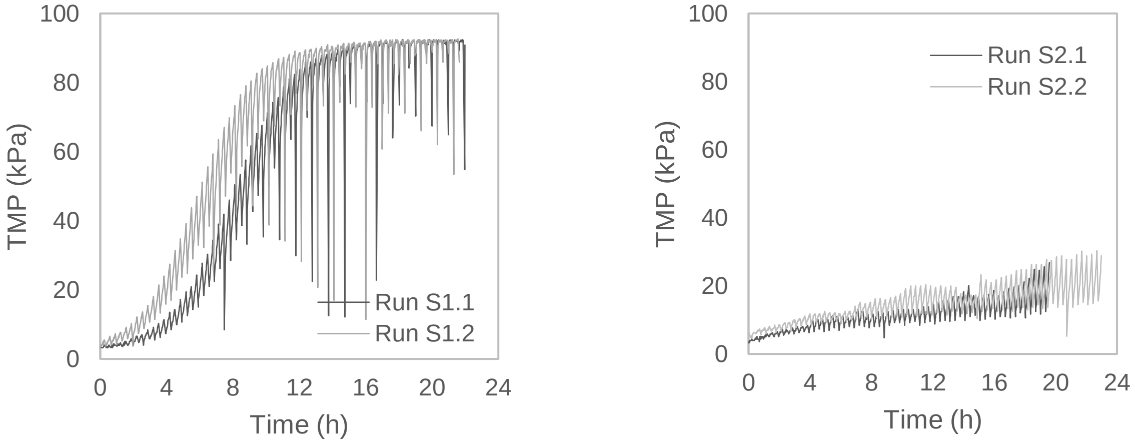

(**a**) (**b**)

Figure S1. The transmembrane pressure evolution during the operation of Runs **(a)** S1 without granular scouring and **(b)** S2 without CEB.

C Assessment of Organic Carbon Recovery by the HR-MBR

Carbon recovery in the operation of the HR-MBR was assessed based on the mass balance as follows:

$$\text{Inflow} - \text{Outflow} - \text{Accumulated} - \text{Transformed} = 0 \qquad \text{(S1)}$$

where inflow is the influent; outflow includes the effluent, sampling, sensor cleaning and maintenance losses, and the concentrate (product); accumulated includes the suspension in the tank, the deposited mass at the bottom of the tank, the deposition on the membrane surface and the deposition formed on tank walls and granules; and transformed refers to mineralization. In this study, the deposits on the tank walls, membranes and granules and the loss associated with maintenance were attributed to losses of organic matter and treated as part of Loss. Therefore, Equation S1 was modified as follows:

$$Influent - Samples = Concentrate + Permeate + Accumulation + Loss \qquad \text{(S2)}$$

The mass of each component was calculated daily to corroborate the stability of the system. Influent, concentrate and permeate masses were calculated from the composite samples obtained during each 24-hour period and multiplied by the flow rate, and the others were calculated from the concentration of the grab samples collected daily multiplied by the volume.

$$(COD_I \times Q_I) - \frac{COD_{ML}^{f} \times V_s}{t} = (COD_C \times Q_C) + (COD_P \times Q_P) + \frac{\left(COD_{ML}^{f} - COD_{ML}^{i}\right) \times V_R}{t} + LOSS \qquad \text{(S3)}$$

where COD_I, COD_C, COD_P, COD_{ML} refer to the COD in the influent, concentrate, permeate and mixed liquor, respectively. Superscripts "i" and "f" refer to the initial and final concentrations of the mixed liquor during the 24-hour period t (1 day). Q_I, Q_C and Q_P refer to the flow rates of the influent, concentrate and permeate, respectively. V_s and V_R refer to the volumes of the samples and the reactor, respectively.

D Daily Mass Balances

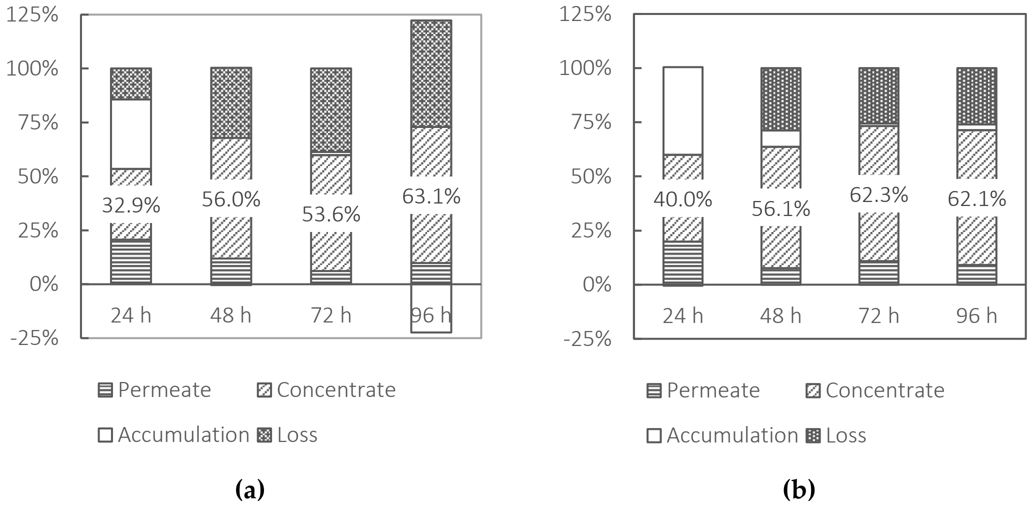

Figure S2. Daily mass balances of the HR-MBR of Run (**a**) 2.1 and (**b**) 2.2.
